# Long Noncoding RNAs as New Architects in Cancer Epigenetics, Prognostic Biomarkers, and Potential Therapeutic Targets

**DOI:** 10.1155/2015/320214

**Published:** 2015-09-13

**Authors:** Didier Meseure, Kinan Drak Alsibai, Andre Nicolas, Ivan Bieche, Antonin Morillon

**Affiliations:** ^1^Platform of Investigative Pathology, Institute Curie, 75248 Paris, France; ^2^Department of Pharmacogenomics, Institute Curie and UMR 745 INSERM, University Descartes, 75248 Paris, France; ^3^UMR 3244, Non-Coding RNA, Epigenetics and Genome Fluidity, Institute Curie, 75248 Paris, France

## Abstract

Recent advances in genome-wide analysis have revealed that 66% of the genome is actively transcribed into noncoding RNAs (ncRNAs) while less than 2% of the sequences encode proteins. Among ncRNAs, high-resolution microarray and massively parallel sequencing technologies have identified long ncRNAs (>200 nucleotides) that lack coding protein function. LncRNAs abundance, nuclear location, and diversity allow them to create in association with protein interactome, a complex regulatory network orchestrating cellular phenotypic plasticity via modulation of all levels of protein-coding gene expression. Whereas lncRNAs biological functions and mechanisms of action are still not fully understood, accumulating data suggest that lncRNAs deregulation is pivotal in cancer initiation and progression and metastatic spread through various mechanisms, including epigenetic effectors, alternative splicing, and microRNA-like molecules. Mounting data suggest that several lncRNAs expression profiles in malignant tumors are associated with prognosis and they can be detected in biological fluids. In this review, we will briefly discuss characteristics and functions of lncRNAs, their role in carcinogenesis, and their potential usefulness as diagnosis and prognosis biomarkers and novel therapeutic targets.

## 1. **Long Noncoding RNAs and Functional Organization of the Genome**

### 1.1. Genome and Noncoding RNA

Large-scale genomic technologies (high-resolution microarray, whole genome, and RNA sequencing) combined with bioinformatics analyses have profoundly changed the genome organization understanding. Unexpectedly, these global transcriptional analyses revealed that 66% of the genome is transcribed and 80% presents biochemical marks of active transcription whereas less than 2% encodes proteins [1]. Discovery of a “second genetic code” consisting of ncRNAs has changed traditional concept of genomic organization characterized by presence of genes encoding islets scattered in a sea of repeats and nontranscribed proteins. Recently, high-throughput techniques have identified 58648 human lncRNAs [2].

### 1.2. Classification of Noncoding RNAs

NcRNAs are classified into two categories according to their structural or regulatory properties and size. This length, arbitrarily set to 200 nucleotides (nt), corresponds to the threshold of sensitivity of RNA extraction methods and can differentiate lncRNAs from short and medium ncRNAs such as microRNAs (miRNAs), small nucleolar RNAs (snoRNAs), and piwi RNAs (piRNAs) [3] (Table 1). Although functions of most ncRNAs are currently still largely uncharacterized, recent studies have involved them in mechanisms implicated in important biological functions and in various pathologic conditions, including neurodegenerative diseases and cancer [4, 5]. It is likely that development of new diagnostic and prognostic classifications involving ncRNAs refine medical practice and that ncRNAs may be useful potential targets for novel anticancer therapies. In this review, we propose to summarize recent literature data on lncRNAs, their involvement in carcinogenesis, and their value as biomarkers and potential therapeutic targets.

### 1.3. Definition of lncRNAs

LncRNAs transcripts are defined by a length greater than 200 nts without any potential of translation [6]. This definition is however arbitrary and cannot now be based on a set of physical biochemical, structural or functional criteria. Length is not an absolute criterion because ncRNAs smaller than 200 nts (*BC1, snaR*) are included in the lncRNAs subgroup. There is no specific transcription of lncRNAs, the transcriptional machinery being common to lncRNAs and mRNAs. Lack of reading frame cannot also be a discriminating factor between mRNAs and ncRNAs because 50% of lincRNAs contain regions with high translational potential, comparable to those of mRNAs, suggesting that they are actively exported to cytoplasm and translated into short polypeptides, albeit certainly not active. Noncoding characteristic is then not specific to lncRNAs, as bifunctional coding and noncoding lncRNAs and lncRNAs containing other ncRNAs (miRNAs, snoRNAs) have recently been discovered. Several mRNAs lose their ability to encode proteins (*Xist*) while others acquire coding function (*SRA*) [7]. Furthermore, by incorporating multiple exons from coding and noncoding genes, alternative splicing generates ambiguous transcripts beyond all classifications. Nevertheless, this definition appears currently to be the best that could be used waiting for a functional characterization of lncRNAs.

### 1.4. Investigative Methods and Annotation of lncRNAs

The first lncRNAs were discovered by using functional genetic approaches based on their relation to specific cellular mechanisms: (i)* Ubxn* transcriptional interference-induced repression by trithorax-promoted lncRNA*bdx*, (ii) sex chromosome dosage compensation and* Xist*-induced X chromosome, and (iii) inactivation and genomic imprinting promoted by antisense lncRNAs* Airn* and* Kcnq1*. These seminal studies established the concept of functional lncRNAs acting as transcriptional regulators involved in important and diverse biological processes. Over the past two decades, systematic global sequencing of cDNA libraries yielded comprehensive knowledge of the transcribed but noncoding component of the genome. These transcriptomic analyses have identified numerous lncRNAs. RNA immunoprecipitation and siRNA-induced inactivation techniques led to functional validation of many lncRNAs.

Currently, lncRNAs identification and functional characterization are based on an experimental approach combining (i) discovery of new noncoding transcripts using RNA-seq and ChIP-seq, (ii) annotation of transcribed regions extent and quantity of products transcribed through bioinformatic analysis, (iii) lncRNAs quantification in various cellular and tissue types and conditions, (iv) coexpression or coregulation studies, and (v) gain and loss functional tests on cell lines and xenografts (siRNAs, ASO, TALEN, and CRISPR/CAS9). Advantage of RNA-seq technique resides in its sensibility, allowing most weakly expressed lncRNAs to be identified. However, due to their frequent very low expression and complex exon/intron structures, it can be difficult to identify different transcripts produced from alncRNA gene. It is then necessary to use other techniques through epigenetic analyses (markers of promoter regions or entire gene), targeted transcripts of interest sequencing after capture on a DNA chip (capture-RNAseq) and 5′ and 3′ extremities identification analyses [8–11].

Systematic large-scale projects have contributed a lot to the systematic characterization of mammalian lncRNAs. For instance, the Fantom (Functional Annotation of the Mammalian genome) is an international project initiated by Japan in 2000 designed to identify and annotate entire transcripts of mouse. Results published identified 15000 lncRNAs in 2002, 23000 in 2005, and 34000 in 2006 with a transcribed genome fraction of 63%. The ENCODE project initiated in 2003 is designed to identify, map, and make public all functional elements of the human genome. This project led to a catalog of lncRNAs with 10000 genes producing 15000 lncRNAs. Very recently, 58648 lncRNAs were identified by using the TCGA database, of which 79% were previously unannotated [2].

### 1.5. Properties of lncRNAs

LncRNAs genes outnumber those of short ncRNAs and are probably more abundant than mRNA genes. LncRNAs are very heterogeneous in size with some extending over tens of kilobases (kb). They are transcribed in all regions of eukaryotic genome, particularly during development, and are remarkably specific for a given cellular and tissue type.

LncRNAs genes share many biochemical characteristics with proteins coding genes: predominant action of RNA polymerase II, epigenetic profiles, cotranscriptional modifications (5′Cap, pre-lncRNAs alternative splicing, and 3′ polyadenylation), and exons and introns size. However, when lncRNAs are compared to mRNAs, they appear less stable and shorter, with fewer exons, a less well defined reading frame, and many repeat sequences (LINE, SINE). A minority group of poorly characterized lncRNAs is represented by nonpolyadenylated lncRNAs synthesized by RNA polymerase III and lncRNAs synthesized via alternative splicing and snoRNAs.

Depending on their relative position to the nearest coding genes, several categories of lncRNAs can be identified as follows.

*(i) Intragenic Regions*. Intragenic IncRNAs may be subdivided depending on how they overlap protein-coding genes or their orientation [45].

- Long intronic ncRNAs (linRNAs) constitute the major component of ncRNAs transcriptome. Unlike lincRNAs, tens of thousands linRNAs have recently been identified but only few have been analyzed functionally [46].
- Sense lncRNAs are transcribed from the sense strand of protein-coding genes containing exons. They may have some overlap with coding genes or cover entire sequence from an intron. If it has been shown that most have no protein-coding potential, several sense lncRNAs can function as both ncRNAs and protein-coding genes (*SRA, ENOD40*).
- Antisense lncRNAs (NAT) represent 32% of lncRNAs in humans and are poorly defined. They are transcribed from the antisense strand of protein-coding genes coding. They may also have overlapping with exonic or intronic regions or cover entire sequence from an intron. NATs are observed in many species, including animals, plants, yeasts, prokaryotes, and viruses, but have no sequence or conserved structure that could be indicative of a particular function despite several highly conserved lincRNAs [47].

*(ii) Intergenic Regions*.* Intergenic lncRNAs (lincRNAs)* are located in unannotated genomic regions and represent the best studied class of lncRNAs. LincRNAs are functional lncRNAs characterized by stability, K4H36 active domain of transcription, and tissue-specific expression. They act in trans and are primarily involved in epigenetic regulation of protein-coding genes expression and maintenance of stem cells pluripotency [48].

*(iii) Enhancer ncRNAs (eRNAs).* Two types of lncRNA (multiexonic or lacking introns with bidirectional transcription) are derived from enhancer sequences. Enhancer sequences regulate temporal and specific expression of genes via cis or trans mechanisms. The human genome contains approximately one million enhancer sequences most likely controlling the 19000 genes coding for proteins. LncRNAs derived from enhancer sequences play a crucial role in the formation of chromatin loops that allow stabilization and association of enhancer sequences with the promoter regions that initiate transcription of their target genes [49].

*(iv) Promoters*. PALR (promoter associated long ncRNA).

*(v) Telomeric Regions.* TERRA (telomeric repeat containing RNAs).

Within cells, lncRNAs are located in the nucleus, according to their main protein-coding genes epigenetic regulatory function. A minority of lncRNAs are involved in nucleocytoplasmic trafficking (*NRON*), mRNA stability, and translational regulation. Several lncRNAs (*MALAT1*) are cleaved by RNase P in a structural and regulatory intranuclear component (speckle) and a small cytoplasmic tRNA-like transcript. LncRNAs topography recognition should help in finding new efficient lncRNAs-based targeted therapies [50]. Finally, lncRNAs can be precursors of ncRNAs (snoRNA, miRNA).

### 1.6. Modular Organization of the Genome and Structural Plasticity of lncRNAs

The genome has a modular architecture composed of complex transcriptional loci characterized by close links between nucleotide sequences organized in sense/antisense and coding/noncoding transcripts. Thus, over 50% of protein-coding genes are associated with complementary antisense noncoding transcripts cis-regulating chromatin and adjacent genes expression although no systematic study classified them carefully as lncRNAs. Moreover, combined application of alternative splicing and transcriptional initiation and termination utilizes this modular architecture to ensure transcriptional diversification illustrated by the very large number of lncRNAs isoforms [48]. LncRNAs structural plasticity and biochemical properties better explain the great diversity of their mechanisms of action than their mere nucleotide sequence. Their organization in secondary and tertiary structures contributes to the creation of functional domains interacting with proteins (small ligands, multiprotein complexes) or hybridizing with nucleic acids (mRNA, miRNA, and DNA). Their interactions lead in turn to lncRNAs allosteric conformational changes allowing them to bind to other actors involved in gene expression and mRNA translation. Thus, lncRNAs initiate regulatory networks with high complexity at epigenetic, transcriptional, and posttranscriptional levels, in order to transmit and coordinate information flows in signaling pathways required for eukaryotic cells functioning [51].

### 1.7. Conservation, Evolution, and Origin of lncRNAs

LncRNAs have been observed in many eukaryotes. Conversely to protein-coding sequences, lncRNAs have rapidly evolved, explaining that lncRNAs orthologues to mammals are only found in vertebrates. Even in vertebrates, DNA sequences conservation is low and suggests that most lncRNAs are not functional due to insufficient selection pressure. Nonetheless, others criteria could be taken into account in assessment of their conservation, including genomic localization, transcriptional profile, and tertiary structure.

Current complexity of human physiology cannot be solely explained by expression of 20000 protein-coding genes, comparable to that observed in Drosophila melanogaster and Caenorhabditis elegans, but rather by parallel development of a noncoding genome. Unlike proteome, amount of ncRNAs has increased during evolution and it seems to be a significant correlation between lncRNAs expression levels and complexity, explaining that primates have most lncRNAs. The number of protein-coding genes cannot explain functioning of very finely regulated organs such as brain. This additional level of complexity observed in vertebrates may be partly related to expression of lncRNAs with a high spatiotemporal specificity and their interactions with DNA, mRNAs, and proteins. LncRNAs could then be considered as molecular sensors of environmental changes conferring evolutional plasticity that contributes to development of life complexity. During environmental changes, lncRNAs located within intergenic sequences may serve as supports for new functions allowing body to adapt new constraints. Protein-coding genes could generate transcripts from lncRNAs genes. Conversely, proteins may be synthesized from lncRNAs. Recent data have identified 24 human protein-coding genes with noncoding homologous genes in other species.

Recent emergence of numerous lncRNAs suggests that they continue to be actively synthesized (i) from ancestral genes that have lost their coding potential, (ii) by genes or other lncRNAs duplication, transposition, and mutation and (iii) de novo from intergenic DNA [52, 53].

### 1.8. LncRNAs Functions and Mechanisms of Action

Growing data have demonstrated that lncRNAs exhibit the greatest diversity among functional ncRNAs and play regulatory and structural roles in embryogenesis, stem cells pluripotency, allelic expression, protein-coding genes regulation, apoptosis, cycle control, growth, differentiation, and senescence. In practice, only a very limited number of lncRNAs (1%) have been well characterized functionally in humans, including* Xist, KCNQ10T1, AIR, HotAir, ANRIL, HOTTIP, MALAT1, TERRA, and HULC.*

LncRNAs play a role in stem cells and differentiation: maintenance of pluripotency and lineage differentiation in embryonic stem cells (ESCs) and induced pluripotent stem cells (iPSCs). Adult stem cells are finely regulated by lncRNAs networks that are targets of most of master pluripotency transcription factors (Oct4, Sox2, Nanog, cMyc, and KLF4) [54].

LncRNAs are considered as crucial regulators coordinating protein-coding genes expression by numerous mechanisms depending on their cellular localization and leading to modifications of chromatin, transcription, and translation.

These regulatory mechanisms are located at epigenetic, transcriptional, posttranscriptional, and translational levels as follows.

(i) LncRNAs constitute a network of epigenetic modulators, recruiting, guiding, and forming platforms that build ribonucleoprotein complexes at specific genomic sites. 20% of human lncRNAs recruit multimolecular repressor, activator, or chromatin remodeling complexes (PRC1/PRC2 by* ANRIL*, LSD1/PRC2 by* HOTAIR*) whose subcatalytic units (EZH2, EED, BMI1, SUZ12, CBX7, CoREST, and JARID1) interact by altering histone code and methylation profile. Cis-acting lncRNAs adjacent to locus where they are transcribed act by transcriptional interference or by modifying chromatin. Transcriptional interference allows inhibition of preinitialization complexes and interaction with transcription factors. Chromatin modifications result from recruitment of complexes inhibiting (Polycomb by* ANRIL*) or activating (MLL by* HOTTIP*) gene expression. Trans-lncRNAs operate independently of complementarities sequences and act at distance on many genomic loci via specific DNA motifs. They influence genes expression by recruiting modifying chromatin complexes, binding transcriptional elongation factors, and inhibiting RNA polymerase [55].

(ii) LncRNAs associated with promoters (PaRNAs) and lncRNAs of enhancer type (eRNAs) can directly regulate transcription of target genes by transcriptional activation or suppression. PaRNAs function as protein coactivators facilitating gene transcription. PaRNAs of 50–200 nt are involved in repression of polycomb-targeted genes via mechanisms allowing transcription of these lncRNAs from common promoter regions to those of target genes regulated by these noncoding transcripts. Gene cis-repression is the result of PRC2 complex (SUZ12) recruitment by PaRNAs, attachment to promoter regions, and modification of histone methylation (H3K27me3) on target promoters. ERNAs have transcriptional activation functions of protein-coding genes coding, including genes involved in embryonic development and differentiation (*ncRNAa3 and TAL, ncRNAa7 and SNAI1*) [56–58].

(iii) LncRNAs can also act at posttranscriptional level and are widely involved in biogenesis, stability, and transcriptional activity of mRNAs. They regulate alternative splicing, promote trafficking, direct cellular localization, and promote mRNAs degradation. LncRNAs also synthesize miRNAs and build sponge-like structures to prevent binding of miRNAs to their target mRNAs (*CDR1-as/ciRS-7*,* circular RNA sponge for miR-7*) [59].

(iv) LncRNAs can finally bind to inhibiting factors of translation, interact with ribosomes, and allow transport of proteins.

LncRNAs may participate in assembly of specialized intranuclear functional structures, including speckles (*MALAT1*) paraspeckles (*NEAT1*), and polycomb body (*TUG1*).

Several lncRNAs, particularly lincRNAs, interact with factors (Oct4, Sox2, Nanog, c-Myc, Klf4, Smad, and Tcf3) and play key roles in maintaining stem cells pluripotency.

**Figure 1:**
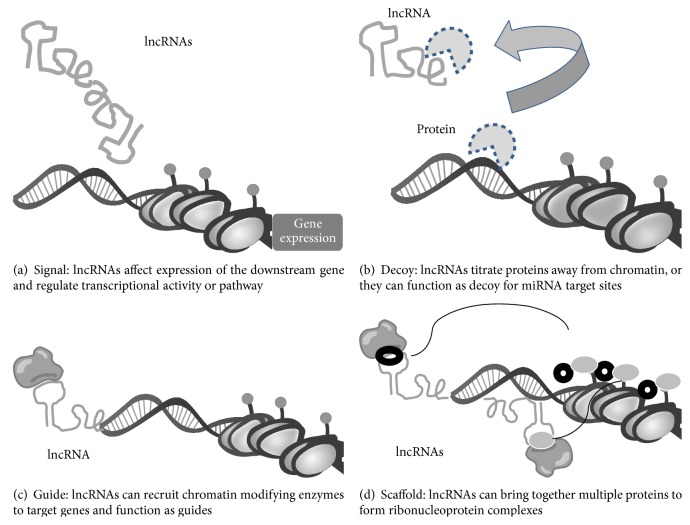
Molecular functions of lncRNAs.

Moreover, lncRNAs have four known molecular functions (Figure 1): (1) Signal: lncRNAs regulate transcriptional activity or pathways (lincRNA-p21); (2) Guide: lncRNAs link specific proteins belonging to chromatin remodeling complexes and recruit them to homology containing target loci where genes silencing is promoted (HOTTIP, XIST); (3) Decoy: lncRNAs bind and titrate away proteins or RNAs. In the nucleus, they can bind transcription factors or DNA methyltransferase 1 (PANDA, Gas5, MALAT1), whereas in the cytoplasm, they can function like a sponge to attract proteins and miRNA/RISC complexes from their miRNA targets; (4) Scaffold: lncRNAs constitute adaptors that bind molecular complexes and regulate gene expression (HOTAIR, ANRIL, and TERC/TERT) [18, 60].

### 1.9. lncRNAs and Cancer

Because lncRNAs are involved in various and important physiological processes, their dysfunction should have important consequences for cell homeostasis. Several recent studies have indeed shown that expression of many lncRNAs varies significantly in different conditions compared to healthy tissue. Significance of these deregulations (consequence of a globally altered transcriptional status or causative and driving abnormality) is still a matter of debate. However, transcripts of noncoding genome revealed new dimension of the molecular architecture of cancer and lncRNAs are involved in all stages of oncogenesis. Gene expression profiles analysis of various tumors showed that lncRNAs are deregulated and functional studies have demonstrated that lncRNAs are implicated in general mechanisms of carcinogenesis. Moreover, genetic studies have revealed existence of mutations in their primary sequences. Since most genetic variants identified by genome-wide association studies (GWAS) are located outside coding genes, many of these mutations may therefore affect lncRNAs.

LncRNAs may regulate signaling pathways involved in initiation, tumor progression, and metastatic spread similar to protein-coding oncogenes and oncosuppressors.* PINC* and* PGEM1* were the first oncogenic lncRNAs found overexpressed in breast and prostate carcinomas. Since then, many other lncRNAs have been identified, with oncogenic properties (*KRASP, HULC, HOTAIR, MALAT1, HOTTIP, ANRIL,* and* RICTOR*) or oncosuppressive properties (*MEG3, GAS5, LincRNA-p21, PTENP1, TERRA, CCND1/CyclinD1, *and* TUG1*). Interestingly, several lncRNAs may show both oncogenic and oncosuppressive activities, depending on cellular context.* XIST* noncoding transcript is overexpressed in male tumors and underexpressed in female tumors.

*(i) Oncogenic lncRNAs*

- *SRA* (steroid receptor RNA activator) is a coactivator for steroid receptors and acts as an ncRNA found in the nucleus and cytoplasm. SRA regulates gene expression mediated by steroid receptors through complexing with proteins also containing steroid receptor coactivator 1 (SRC-1). The SRA1 gene can also encode a protein that acts as a coactivator and corepressor. SRA levels have been found to be upregulated in breast tumors where it is assumed that increased SRA levels change the steroid receptors' actions, contributing to breast carcinogenesis. While expression of SRA in normal tissues is low, it is highly upregulated in breast, uterus, and ovary carcinomas [61–63].
- *HOTAIR* (HOX antisense intergenic RNA) is a long intergenic noncoding RNA with a length of 2.2 kb in* HOXC* locus and transcribed in antisense manner. HOTAIR regulates gene expression by modulating chromatin structures. It was the first lncRNA discovered to be involved in carcinogenesis. Polycomb group proteins mediate repression of transcription of thousands of genes that control differentiation pathways during development, pluripotency, and cancer progression. Target of PRC2 is* HOXD* locus on chromosome 2 where PRC2 in association with* HOTAIR* promotes transcriptional silencing of metastasis suppressor genes.* HOTAIR* acts as a molecular scaffold remodeling chromatin by causing histone modifications on target genes.* HOTAIR* comprises two known chromatin modification complexes with 5′ region binding to PRC2 complex responsible for repressive H3K27 methylation and 3′ region binding to LSD1, which initiates activating H3K4 demethylation [64, 65].
- *ANRIL* (antisense ncRNA in the INK4 locus) is a natural antisense transcript, which activates the two polycomb repressor complexes PRC1 and PRC2, resulting in chromatin reorganization with silencing of* INK4b-ARF-INK4a*. This results in p15/INK4b, p14/ARF, and p16INK4a inhibition which are normally implicated in cell cycle negative regulation, senescence, and stress-induced apoptosis.* ANRIL* overexpression in prostate carcinomas has shown silencing of INK4b-ARF-INK4a and p15/CDKN2B. Repression mechanism is mediated by direct binding to CBX 7 and SUZ12, 2 members of PRC1 and PRC2, respectively [66–68].
- *MALAT1* (metastasis-associated lung adenocarcinoma transcript 1) is a lincRNA widely expressed in normal human tissues and overexpressed in a variety of breast, colon, liver, pancreas, prostate, and uterus carcinomas.* MALAT1* locus at 11q13.1 has been identified to harbor chromosomal translocation break points and mutations in breast and bladder carcinomas. It is localized in nuclear speckles and recent studies have implicated* MALAT1* in splicing, EMT, cell mobility, ECM remodeling, and metastatic spread [69].
- *HULC* (highly upregulated in liver cancer) has been suggested to act as a “sponge” that inhibits miR-372 by sequestering it away from potential mRNA targets [70].
- *PCGEM1* (prostate cancer gene expression marker 1) is overexpressed in prostate cancer cells that promotes tumor cells initiation and progression and protects against chemotherapy-induced apoptosis. Moreover, the reciprocal negative control relationship between PCGEM1 and miR-145 regulates both prostate cancer cells proliferation and tumor growth. These results identify PCGEM1 and associated regulators as possible targets for prostate cancer therapy [71].

*(ii) Tumor Suppressor lncRNAs*

- *MEG3* (maternally expressed gene 3) is a transcript of the maternally imprinted gene normally expressed in pituitary cells.* MEG3* loss of expression is observed in pituitary adenomas and meningiomas.* MEG3* acts by regulating p53 pathway. P53 levels are usually extremely low due to its rapid degradation via ubiquitin-proteasome pathway. P53 ubiquitination is mainly mediated by E3 ubiquitin ligase MDM2.* MEG3* downregulates MDM2 expression, increases p53 protein level, stimulates p53-dependent transcription, and enhances p53 binding to target promoters [72].
- *GAS5* (growth arrest-specific 5) is widely expressed in embryonic and adult tissues.* GAS5* acts as a starvation or growth arrest-linked riborepressor for glucocorticoid receptors by inhibiting association of these receptors with their DNA recognition sequence. This suppresses several responsive genes activation including gene encoding cellular inhibitor of apoptosis 2 (cIAP2).* GAS5* low expression levels have been observed in prostate and breast carcinomas [73, 74].
- *CCND1/Cyclin D1* is transcribed from the promoter region of the Cyclin D1 gene.* Cyclin D1* is a cell cycle regulator frequently mutated, amplified, and overexpressed in carcinomas.* CCND1/Cyclin D1* recruits the RNA-binding protein TLS, which undergoes allosteric modification, resulting in Cyclin D1 gene inhibition [75, 76].
- *LincRNA-p21* expression is directly induced by p53 signaling pathway. This lncRNA is implicated in global repression of genes interfering with p53 function to regulate cellular apoptosis [77].
- *TERRA* (telomeric repeat containing RNA) is expressed at chromosome ends. TERRA upregulation upon experimental manipulation or in ICF (immunodeficiency, centromeric instability, and facial anomalies) patients correlates with short telomeres. TERRA transcription facilitates the 5′-3′ nuclease activity of Exo1 at chromosome ends, providing a means to regulate the telomere shortening rate. Thereby, telomere transcription can regulate cellular lifespan through modulation of chromosome end processing activities [78].

*(iii) Oncogenic and Tumor Suppressor lncRNAs*

- *H19* is expressed from the maternal allele and has a pivotal role in genomic imprinting during cell growth and development. The locus contains* H19* and IGF2, which are imprinted. This leads to differential expression of both genes H19 from maternal and IGF2 from paternal allele. This lncRNA presents both oncogenic and suppressive properties although the exact mechanism is still elusive [79].

LncRNAs are implicated in cancer epigenetics. Epigenetics refers to events that modulate activity of the genome without changing its sequence. It provides control of genome expression and establishment of tissular and cellular-type specific genes expression profiles. The main epigenetic mechanisms comprise chemical modifications of DNA and histones (cytosine methylation, posttranslational modifications of histones, chromatin remodeling, and nucleosome positioning). These chemical changes are reversible and controlled by enzyme complexes directly connected to metabolic and signaling pathways as well as sensors of extra- and intracellular microenvironments. Deregulation of these epigenetic mechanisms has been demonstrated in cancer cells which have no transcriptional gene or protein expression while being free of DNA damage. Carcinogenesis is very frequently associated with abnormal signaling and epigenetic abnormalities, most of which are considered as significant oncogenic events (aberrant methylation of oncosuppressor genes). LncRNAs interact with numerous proteins and in particular epigenetic regulators, including DNA methyltransferases and enzymatic complexes modifying chromatin and nucleosomes [80].

(i) Methylation of DNA was first observed in cancer epigenetic alterations. Cancer epigenome is characterized by global hypomethylation leading to genomic instability and hypermethylation of CpG islands which cause inactivation of genes oncosuppressors implicated in signaling pathways (DNA repair, apoptosis, and cycle cell regulation) or transcription factors involved in the control of these genes. LncRNAs bind to DNA methyltransferase, guide it to promoter of oncosuppressor genes, and ensure transcriptional silencing.

(ii) Nucleosomes can be destabilized and restructured by remodeling complexes belonging to 4 families (SWI/SNF, ISWI, CHD, and INO80). Nucleosomes positioning and remodeling regulate gene expression by perturbating accessibility of DNA regulatory sequences by transcription factors and transcription machinery. Mutations and silencing of subunits of these complexes in various types of cancer have been observed. LncRNAs could reposition nucleosomes by interacting and guiding these remodeling complexes to specific genomic regions where nucleosome restructuring would ensure repression of oncosuppressive genes [80].

(iii) LncRNAs recruit histone modifying enzymes acting near the transcription site of lncRNAs. Histones modifying enzymes read, add, or remove covalent links with change in accessibility of chromatin and fixation of nonhistone protein effectors that decode modified histone code. Alterations in the expression of histone-modifying enzymes (mutations, overexpression) were observed in various carcinomas. These alterations inhibit oncosuppressive genes transcription (EZH2 histone methyltransferase responsible for repressive H3K27me3 mark and silencing of p15, p16, and p19; histone deacetylase responsible for loss of lysine acetylated H4) or activate oncogenes transcription (demethylases and deacetylases) [81].

Growing numbers of LncRNAs are implicated in numerous mechanisms characterizing hallmarks of cancer. They can act on proliferation via coactivation (*SRA1*) and inhibition of elongation (*RN7SK*). LncRNAs allow escape mechanisms from suppressor pathways via competition (*PFS*), silencing (*ANRIL*), or ribonucleic repression (*GAS5*). Several lncRNAs are involved in replicative immortality by inhibiting telomerase (*TERRA*). LncRNAs promote neoangiogenesis is by inhibiting HIF1A (*AHIF, MALAT1*). They induce resistance to cell death by acting on p53 and p21 (*PCGEM1*) and by reducing expression of proapoptotic genes (*PANDA)*. Finally lncRNAs are involved in mechanisms of invasion and metastasis via deregulation of alternative splicing (*MALAT1*) or trans-silencing of HOXD locus (*HOTAIR*).

## 2. LncRNAs as Biomarkers and Therapeutic Targets in Future Medical Practice

LncRNAs are emerging as integral functional components of human genome and are now considered as critical regulators in molecular biology of cancer. Recent data have demonstrated that lncRNAs are associated with cancer initiation, tumor progression, and metastatic spread. Unlike mRNAs used as diagnostic and prognostic biomarkers while being expressed by numerous subtypes of malignant tumors, lncRNAs present cellular and tissular specificity and could serve as biomarkers and therapeutic targets in cancer. Furthermore, lncRNAs secreted or released by apoptotic or necrotic tumor cells can be detected in blood, plasma, and urine. There seems to be a significant correlation between levels of circulating nucleic acids and genomic, epigenetic, or transcriptional alterations associated with malignant tumors (Table 2).

*(i) Esophagus.* Three lncRNA signatures have been identified recently in oesophageal squamous cell carcinoma, including ENST00000435885.1, XLOC_013014, and ENST00000547963.1. The expression of these lncRNAs classified the patients into two groups with significantly different overall survival [12].

*(ii) Stomach. HOTAIR, GCAT1 *(gastric cancer-associated transcript 1),* H19,* and* SUMO1P3* (small ubiquitin-like modifier SUMO 1 pseudogene 3) are the main lncRNAS reported as overexpressed in gastric carcinomas. They are often associated with lymph node and distant metastasis, suggesting that they might serve as potential diagnostic and prognostic biomarker [13–17]. Moreover, CCAT1 expression is closely related to c-Myc activation [14].* CCAT1* functions as an oncogene and may be used as biomarker and potential therapeutic target in gastric carcinoma [15, 16].

*(iii) Lower Digestive Tract. HOTAIR* overexpression is observed in colorectal carcinomas with advanced stage and liver metastases [19]. uc.73 lncRNA is also associated with poor overall survival in patient with colorectal carcinomas [18]. These results place HOTAIR and uc.73 lncRNA as reliable biomarkers for poor prognosis in colorectal cancer.

*(iv) Liver.* In hepatocellular carcinomas (HCC),* HULC* (highly upregulated in liver cancer) was the first lncRNA with highly specific upregulation detected in blood [20]. High plasma* HULC* rates were observed in patients with high grades HCC or with HBV+ status [21]. Recent data have shown that HBx could regulate* HULC* promoter to induce HCC via oncosuppressor* p18* silencing [22]. HBx was found to downregulate lncRNA-*Dreh,* which can inhibit hepatocellular growth and metastasis* in vitro* and* in vivo*.

*MALAT-1* and* HOTAIR* have been shown to be overexpressed in large cohorts of HCC patients. Furthermore,* HOTAIR* is a prognostic biomarker for recurrence after liver transplantation. SiRNA-mediated inhibition of* MALAT-1* and* HOTAIR* suppresses cancer cell viability and invasion, sensitizes TNF-*α*, induces apoptosis, and increases chemotherapeutic sensitivity of HCC to cisplatin and doxorubicin [82, 83].* HOTTIP* (HOXA transcript at the distal tip) and* HOXA13* were also found to be upregulated in HCC.* HOTTIP* and* HOXA13* levels were associated with HCC tumor progression, metastasis, and survival [84].* HEIH* (high expression in HCC) is another lncRNA overexpressed in HCC and an independent prognostic factor associated with recurrence [85].

*(v) Lung. MALAT1* is a key prognostic biomarker for metastatic spread in lung adenocarcinomas [23].* TUG1* (taurine upregulated gene 1) is generally downregulated in non-small cell lung carcinomas (NSCLC). In NSCLC patients,* TUG1* low expression was associated with high TNM stage, tumor size, and poorer overall survival. Univariate and multivariate analyses revealed that* TUG1* expression serves as an independent predictor for overall survival [24].* HOTAIR* was initially reported to be highly expressed in NSCLCs with advanced stage and lymph node metastasis [25]. But more recent meta-analyses study showed that it did not reach statistical significance, and thus it needs further investigations [26]. In NSCLCs,* BANCR* (BRAF activated noncoding RNA) expression is significantly decreased compared to normal tissues.* BANCR* underexpression is considered as an independent prognostic factor and is associated with larger tumor size, advanced pathological stage, metastasis distance, and shorter overall survival. Recently,* BANCR* overexpression was found to play a key role in epithelial-mesenchymal transition [27]. In malignant pleural mesothelioma (MPM),* GAS5* (growth arrest specific transcript 5) underexpression was observed compared to normal mesothelial tissue. Conversely,* GAS5* was upregulated upon growth arrest induced by inhibition of Hedgehog and PI3K/mTOR signaling in MPM models [28].

*(vi) Breast.* Breast cancer progression is correlated with* HOTAIR* activity in numerous recent studies. The study carried out by Chisholm and colleagues demonstrated* HOTAIR* overexpression in primary and metastatic breast carcinoma tissues by using RNA in situ hybridization technique [29]. Another study focusing on* HOTAIR* revealed the dependence of HOTAIR expression on oestradiol production, due to its promoter region via several estrogen response elements. This study also demonstrated that* HOTAIR* knockdown induced apoptotic pathways in breast cancer cell lines and suggested estrogen receptors as coregulators for* HOTAIR* expression [30]. Conversely, a recent study focusing on 348 primary breast carcinomas revealed that increased DNA methylation led to* HOTAIR* downregulation and an unfavorable disease state, questioning suitability of* HOTAIR* as negative prognostic biomarker in breast carcinomas [31].* LincRNA-RoR* suppresses p53 in response to DNA damage through interaction with heterogeneous nuclear ribonucleoprotein I (hnRNP I). Recent data demonstrated that hnRNP I can also form a functional ribonucleoprotein complex with* UCA1* (urothelial carcinoma-associated 1) and increase* UCA1* stability. Of interest, the phosphorylated form of hnRNP I, predominantly located in the cytoplasm, is responsible for the interaction with* UCA1*. Although hnRNP I enhances p27 translation through interaction with the 5′-untranslated region of p27 mRNAs, interaction of* UCA1* with hnRNP I suppresses p27 protein expression via competitive inhibition. In support of this finding,* UCA1* seems to have an oncogenic role in breast carcinogenesis both* in vitro* and* in vivo*. Finally, a negative correlation between p27 and* UCA1 *was found. Together, these results suggest an important role of UCA1 in breast carcinogenesis [32].

*(vii) Glioma. H19*/miR-675 signaling was recently identified as a critical pathway in glioma progression. By analyzing gene expression data,* H19* increased levels of expression were found in high grade glioma. SiRNA-induced* H19* depletion inhibited invasion in glioma cells. Furthermore,* H19* expression was positively correlated with miR-675 and* H19* inhibition reduced miR-675 expression. Collectively, these data suggest that* H19* regulates glioma development by deriving miR-675 [40].

*(viii) ENT Tumors.* Recently,* HOTAIR* was suggested to play a part in nasopharyngeal carcinoma (NPC).* HOTAIR* is implicated in NPC progression and patients with high* HOTAIR* levels have poor clinical outcome with tumor recurrence and distant metastasis [42, 43]. In primary NPCs, upregulation of* lnc-C22orf32-1, lnc-AL355149.1-1, and lnc-ZNF674-1* has been also observed. High levels of* lnc-C22orf32-1* and* lnc-ZNF674-1* are associated with advanced tumor stages. Recurrent NPC displayed a distinctive lncRNA expression pattern with increased expression of* lnc-BCL2L11-3* and decreased expression of* lnc-AL355149.1-1* and* lnc-ZNF674-1* [44]. Interestingly, lncRNAs can be located in whole saliva.* HOTAIR* is differentially expressed in saliva of metastatic oral squamous cell carcinoma patients compared to primary tumors. These findings suggest that detection of lncRNAs in saliva may be used as a noninvasive and rapid diagnostic tool for diagnosis of oral carcinomas [4].

*(ix) Bladder.* Oncogenic lncRNAs, including* UCA1, H19, MALAT1,* and* linc-UBC1* (upregulated in bladder cancer 1), are overexpressed in bladder carcinomas and activate PI3K-AKT and Wnt/*β*-catenin pathways. A pilot study took advantage of this to evaluate potential application of* UCA1* in urinary sediments from patients with bladder carcinomas. It turned out to be especially valuable for superficial G2/G3 patients at a high risk for muscular invasion, indicating that* UCA1* may be a new promising urinary biomarker for the diagnosis of bladder cancer.* H19* expression levels are remarkably increased in bladder carcinomas compared to normal tissue and could also serve as another biomarker. More recently, a new lncRNA* linc-UBC1* was found to be overexpressed in 60% of invasive bladder carcinomas and correlated with lymph node metastasis and poor survival.* MALAT1* is upregulated in bladder cancer and its expression level is correlated with tumor grade and metastatic stage [34, 35].

*(x) Prostate.* Prostate cancer specific lncRNA*DD3/PCA3* was identified fifteen years ago [86].* PCA3* is a prostate-specific lncRNA markedly overexpressed in prostate carcinomas. It can be detected in prostate cancer tissue, urine, and/or urine sediments. In recent studies,* PCA3* specificity was found even higher than prostate biomarker PSA and* PCA3* score can accurately predict tumor volume and pathological features, which may guide treatment [36, 37]. Therefore,* PCA3* can be used as a noninvasive urine-based test for large-scale screening protocols and for predicting prostate carcinomas aggressiveness. This prominent example of rapid translation of lncRNA research into clinical practice offers a prototype for developing different lncRNAs as biomarkers. Other lncRNAs, such as PCGEM1 and PCAT1 (prostate cancer associated ncRNA transcript 1), are also prostate-specific, posing as attractive biomarkers [38, 39].

*(xi) Melanoma. BANCR *(lncRNA BRAF-activated noncoding RNA) plays a potentially functional role in melanoma cells proliferation and migration by activating ERK1/2 and JNK MAPK pathways.* BANCR* is upregulated in human malignant melanoma and patients with high levels of expression have a lower survival rate [41].

*(xii) Hemopathies.* Several recent reports have revealed deregulation of lncRNAs in leukemia, including* ANRIL, lncRNA-P21, MEG3, Dleu2, HOTAIRM1, EGO*, and* lncRNA-a7*. Moreover,* MEG3, UCA1,* and* H19* are upregulated in acute myeloid leukemia [87, 88].

*(xiii) Ovary. HOTAIR* plays a pivotal role in epithelial ovarian cancer (EOC) metastasis and could represent a novel prognostic marker and potential therapeutic target in patients with EOC. In recent study,* HOTAIR* expression was elevated in EOC tissues, and its level of expression is highly positively correlated with FIGO stage, histological grade, lymph node metastasis, reduced overall survival, and disease-free survival [33].

Because of their central role in genes expression regulation, lncRNAs could also represent potential therapeutic targets. Better characterization of lncRNAs (structure, functions, polymorphisms, and intracellular topography) could help in faster development of new anticancer strategies modulating expression levels and functions of deregulated lncRNAs. Targeting lncRNAs offers novel exciting opportunity to treat cancer. Currently, nucleic acid-based methods prevail in targeting RNA, by regulating levels of expression and modifying their structures or mature sequences. Among them, RNA interference (RNAi) based techniques are arguably the most popular methods to inhibit lncRNAs in cancer cells. Both siRNAs and shRNAs exhibit great RNA selectivity and knockdown efficiency. Meanwhile, other established methods in inhibiting cancer-associated RNA, including antisense oligonucleotide (ASO), ribozyme, and aptamer, are also effective to modulate lncRNAs, and they show unique features that can have advantages over siRNAs [89].

In principle, targeting of lncRNAs can be achieved using the several approaches, including siRNA-mediated silencing, functional block using small molecules, or oligonucleotide inhibitors to prevent interactions of lncRNAs with proteins and structure disruption via small molecules or oligonucleotide inhibitors to change or mimic their secondary structure to compete for their binding partners [90].

(1)* Small interfering RNAs (siRNA)* are short stretched (19–30 nt) double-stranded RNAs that target RNA molecules via complementary to unpaired lncRNA sequences. The RNA duplex of siRNAs must be unwound into single strands before assembling into the active RNA-induced silencing complex (RISC). SiRNAs are fully complementary to their RNA target that is then cleaved at a single phosphodiester bond located near the centre of the sequence complement to the siRNA sequence [90]. SiRNAs exhibit high knockdown efficiency to many oncogenic lncR-NAs in cancer cells and induce anticancer effects both* in vitro* and* in vivo*. For instance, depletion of* HOTAIR* by siRNAs decreases matrix invasiveness of breast cancer cells and inhibits tumor growth of pancreatic cancer xenograft.* HULC* and* MALAT1* siRNA-induced knockdown inhibits HCC cell proliferation and cell cycle progression [91]. Furthermore, siRNA-mediated knockdown of* H19* induces apoptosis and inactivates p53 [92]. Phase I and II clinical therapeutic trials have been evaluated using siRNAs to inhibit critical cancer-associated genes, including siRNA-EphA2-DOPC (targeting* EphA2*), TKM-080301 (targeting* PLK1*), and CALAA-01 (targeting* RRM2*). Therapy combining siRNA drug siG12D LODER and Atu027 with conventional chemotherapy is studied in phase II for its therapeutic effect in advanced pancreatic cancer patients. However, the main obstacle of siRNAs therapeutics remaining is their delivery. SiRNAs using ribonucleic acid as building block are susceptible to be degraded by nuclease and have poor pharmacokinetics.

(2)* Antisense oligonucleotides (ASO)* are short, single-stranded DNAs or RNAs (between 8 and 50 nt) designed with sequence specific to target lncRNA [90]. ASOs directly hybridize to lncRNA transcripts via base pairing and endogenous RNase H1, which results in cleavage of lncRNA molecules, then recognizes hybrids formed. ASOs modulate lncRNAs function through degradation of lncRNA transcripts. Inhibition of* MALAT1* by ASO attenuates various malignant phenotypes in cancer cells via cycle arrest in cervical cancer cells [93]. Injection of ASO into subcutaneous tumors of nude mice effectively inhibits* MALAT1 in vivo* and blocks metastasis of lung cancer cells [91].

(3)* Ribozymes* are naturally produced RNA molecules that present intracellular catalytic functions. One of their functions is degradation of RNA molecules. Among all types of ribozyme, hammerhead ribozyme (HamRz) has caught major interest as it shows good target inhibitory effect while having the smallest RNA endoribonucleolytic motif as well as function independent to presence of metal ions [94]. HamRz is single-stranded RNA in neutral condition and undergoes folding in cells to expose the binding arms. Binding of HamRz to target sequence depends on complementary match with homologous target site. Both arms of HamRz have to bind with target sites correctly in order to form functional catalytic motif. After binding, HamRz catalyzes cleavage of the flanked RNA region downstream to a NUH site via destabilizing phosphodiester backbone of target RNA [90].

(4)* Aptamers* are short DNA or RNA oligonucleotides or peptides that have a stable 3-dimensional structure* in vivo*. They have broad molecular targets including protein, RNA, and small molecules that rely on fitting 3-dimensional shape of their ligands [95]. They specifically bind to their target lncRNAs that rely on fitting 3-dimensional shape of the lncRNA structures. Aptamers antagonize their lncRNA targets by blocking the interactions between lncRNAs and critical factors [90]. Some reports show promising effects of aptamers to either degrade RNA or inhibit RNA functions, implying the potential of aptamers as therapeutic agents to target lncRNAs. Aptamers are used to modulate viral gene expression by interacting with viral RNAs. A hairpin aptamer is identified to form stable and specific complex with the transactivation response element (TAR) RNA element of HIV-1 mRNA and decrease the TAR-dependent viral protein expression [96]. Another aptamer, selected against TAR element, formed stable loop–loop complexes with the element to regulate the TAR-mediated process [97]. Another study showed that aptamer could target the apical loop domain of pri-microRNA molecules and modulate the biogenesis of mature microRNA [98].

(5)* MicroRNAs (miRNAs)* can interact with lncRNAs.* H19/*miR-675 signaling is critical in glioma progression. By analyzing glioma gene expression data sets,* H19 is* found to be increased in high grade gliomas.* H19* depletion via siRNA inhibits invasion in glioma cells. Further, H19 is positively correlated with its derivate miR-675 expression and reduction of* H19* inhibits miR-675 expression. MiR-675 modulates cadherin 13 expression by directly targeting its binding site within 3′ UTR. These results demonstrate that* H19* regulates glioma development by deriving miR-675 and provide important clues for understanding key roles of lncRNA-microRNA functional network in glioma. MicroRNA-based anticancer therapy has great potential as reports show there is apparent lack of adverse event in normal tissues when administrated with microRNA-based agent. Various microRNA delivery strategies, such as cationic lipid and nanoparticle encapsulation, are developed to improve microRNA shuttling into target cancer cells [40, 99, 100]. The potential of microRNA regulation on lncRNAs can be further realized by better understanding of intragenic lncRNA regulating elements.

(6)* Small molecules* are synthesized to specifically bind to RNA binding pockets of lncRNAs. They compete with protein factors or intracellular small ligands for binding lncRNAs. Binding of small molecules may also induce conformational change within lncRNA molecules and disrupt formation of important lncRNA structures [90].

## 3. Conclusion and Perspectives

Deregulated oncogenic and oncosuppressive lncRNAs are observed at all stages of malignant tumors development of various origins. Mechanisms implicating lncRNAs in carcinogenesis are dominated by deregulation of signaling pathways and altered epigenetic, transcriptional, and posttranscriptional expression of numerous genes. Their use as biomarkers and potential therapeutic targets could appear promising.

Major challenges of the next 10 years remaining are identification, mapping of all lncRNAs belonging to the human genome, and their functional characterization. Although their mechanisms of action are better known, it is still possible that lncRNAs are rather downstream products of particular chromatin structures or deregulated transcriptional processes. Their functionality is currently still debated because of their low level of expression and conservation between species. Unlike proteins, which often have well-defined functional areas, it is currently impossible to predict lncRNAs function from their single sequence. However, many traits exhibited by lncRNAs favor functionality, including 3′polyadenylation, 5′cap, multiple exons, transcriptional activation similar to that of mRNA, K4K36 domain, and alternative splicing. These challenges will only be achieved through combined efforts of functional genomics, epigenomics, and bioinformatics.

## Figures and Tables

**Table 1:** Type and main function of no-coding RNAs.

Type	Size (nt)	Functions
Short ncRNAs	19–31	
miRNAs		Targeting of mRNAs, regulation of proliferation, differentiation, and apoptosis involved in human development
siRNAs		Posttranscriptional gene silencing; defense against pathogenic nucleic acids
tiRNAs		Regulation of transcription by targeting epigenetic silencing complexes
piRNAs		Transposon repression, DNA methylation, development of germ cell, stem self-renewal, and retrotransposon silencing
tel-sRNAs		Epigenetic regulation
Mid-size ncRNAs	≤200	
snoRNAs		rRNA modifications
PASRs		Regulation of the transcription of protein-coding genes
TSSa-RNAs		Maintenance of transcription
PROMPTs		Activation of transcription
crasiRNAs		Recruitment of heterochromatin and/or centromeric proteins
Long ncRNAs	>200	
lincRNAs		Involvement in biological processes such as dosage compensation and/or imprinting
Intronic lncRNAs		Possible link with posttranscriptional gene silencing
T-UCRs		Regulation of miRNA and mRNA levels and antisense inhibitors for protein-coding genes or other ncRNAs
TERRAs		Negative regulation of telomere length and activity through inhibition of telomerase
Pseudogene RNAs		Regulation of tumor suppressors and oncogenes by acting as microRNA decoys
lncRNAs with dual functions		Modulate gene expression through diverse mechanisms

**Table 2:** Representative lncRNAs involved in carcinogenesis and potential cancer biomarkers.

Cancer type	lncRNAs	References
Esophagus	*ENST00000435885.1, XLOC_013014, ENST00000547963.1 *	[12]
Stomach	*GCAT1, H19, SUMO1P3 *	[13–17]
Colon and rectum	*HOTAIR, uc.73 *	[18, 19]
Liver	*HULC, HOTAIR, MALAT1, HOTTIP, HEIH *	[20–22]
Lung	*MALAT1, TUG1, BANCR, GAS5 *	[23–28]
Breast	*HOTAIR, LincRNA-RoR, UCA1 *	[29–32]
Ovary	*HOTAIR *	[33]
Bladder	*UCA1, H19, Linc-UBC1, MALAT1 *	[34, 35]
Prostate	*PCA3, PCAT1, PCGEM1 *	[36–39]
Glioma	*H19 *	[40]
Melanoma	*BANCR *	[41]
Oral cavity and nasopharynx	*HOTAIR, lnc-C22orf32-1, lnc-AL355149.1-1* *lnc-ZNF674-1 *	[4, 42–44]
